# Longitudinal trajectories of empathy among chinese medical students: a five-year prospective study using a dual-scale assessment

**DOI:** 10.1186/s12909-026-09135-5

**Published:** 2026-04-01

**Authors:** Dongju Li, Jiaqi Liu, Huiming Xu, Shulan Ma

**Affiliations:** 1 Experimental Teaching Center of Basic Medical Science, School of Basic Medical Sciences, Fudan University, P.O. Box 117., 138 Yi-Xue-Yuan Road, Shanghai, 200032 China; 2 Student Affairs Office, School of Basic Medical Sciences, Fudan University, P.O. Box 275., 138 Yi-Xue-Yuan Road, Shanghai, 200032 China; 3 Nanjing West Road Community Health Service Center, 165 Cheng-du North Road, Shanghai, China

**Keywords:** empathy, Chinese medical students, longitudinal study, Jefferson Scale of Empathy, Interpersonal Reactivity Index

## Abstract

**Background:**

Empathy is a core competence in medical practice that contributes to patient outcomes and physician well-being. Although widely studied, the developmental trajectory of empathy during medical education remains unclear, particularly in China. To date, no long-term longitudinal studies have examined empathy development among Chinese medical students.

**Methods:**

We conducted a five-year prospective longitudinal study among medical students at Fudan University. Both the Jefferson Scale of Empathy–Student version (JSE-S), which assesses context-specific clinical empathy in medical settings, and the Interpersonal Reactivity Index (IRI), which measures dispositional (trait) empathy in general social contexts, were administered annually from 2019 to 2023. Mixed-effects models were used to analyze changes in empathy and their predictors, with fixed effects including academic year, age, career aspiration, and entry year.

**Results:**

A total of 104 students contributed 266 valid responses across multiple measurement waves. JSE-S scores significantly decreased over time, with fifth-year scores markedly lower than first-year scores (B = − 11.57, *p* < .001). Older students and those with stronger medical career aspirations reported higher JSE-S scores. IRI scores positively predicted JSE-S scores (B = 0.40, *p* < .001) but remained stable across academic years, suggesting that trait empathy was largely unaffected by medical training.

**Conclusions:**

Clinical empathy among Chinese medical students significantly decreased over five years, whereas trait empathy remained stable. These findings highlight the distinct developmental trajectories of clinical and trait empathy and emphasize the value of longitudinal dual-scale assessment. Educational interventions are needed to sustain empathy as a professional competence throughout medical training, particularly in the face of increasing clinical demands and contextual pressures during the later stages of education.

## Background

Empathy is commonly defined as the capacity to perceive, understand, and share the emotions or experiences of others, and it plays a critical role in medical practice. In medical contexts, this construct is conceptualized as clinical empathy, encompassing emotional resonance, cognitive understanding of patients’ experiences, emotion regulation to maintain clinical judgment, and the ability to convey understanding through caring behaviors. The Jefferson Scale of Empathy (JSE) was specifically developed to assess clinical empathy in healthcare settings, particularly its student version (JSE-S) for medical students [1, 2]. In contrast, the Interpersonal Reactivity Index (IRI) evaluates trait empathy, reflecting relatively stable empathic tendencies in general social contexts [3, 4]. Employing both instruments provides a more comprehensive evaluation by capturing context-sensitive clinical capacities alongside more enduring dispositional empathy.

Empathy is increasingly conceptualized as a multidimensional and dynamic process, rather than a single, static trait. Contemporary theoretical models propose that empathy originates from a general empathic capacity that is continuously shaped and modulated by situational and motivational factors [5]. This perspective emphasizes the distinction between the trait and state components of empathy, which has substantially advanced theoretical understanding of empathic processes [6]. Specifically, trait empathy represents a relatively stable individual disposition that determines an individual’s general empathic potential and overall empathic tendency across time and situations, whereas state empathy refers to the context-dependent and momentary expression of empathic responses in specific situations. Importantly, trait empathy and state empathy are interrelated and interact dynamically: trait empathy provides the underlying capacity for empathic engagement, whereas state empathy reflects the actualization of this capacity within a given situational context. This interaction is further modulated by multiple contextual variables, including task demands, attentional allocation, emotional load, as well as individual and sociocultural factors such as gender and culture [7].

Building on this distinction between dispositional and context-dependent empathy, clinical empathy in medical education can be understood as a context-dependent expression of empathic capacity. It is shaped not only by individuals’ empathic dispositions but also by the specific demands and expectations of medical training and clinical environments [8]. Existing research suggests that the trait-state interaction process, together with the contextual regulatory variables discussed above, constitutes a key mechanism influencing empathy development among medical students. Consequently, empathy in medical students is conceptualized as a complex and dynamic process influenced by a range of internal and external factors. Empirical studies demonstrate that individual characteristics (e.g., age, gender, motivation, or career aspirations) [9, 10] and sociocultural background (e.g., collectivist values and norms of emotional expression) [11] can influence the expression of empathy among medical students. Furthermore, demanding educational and clinical environments, high workload, and emotional stress may suppress state empathic responses, whereas supportive learning environments, positive role models, and strong professional motivation may help sustain empathic engagement [8, 12, 13].

Clinical empathy is a core component of professional competence in medicine, contributing not only to improved patient satisfaction, treatment adherence, and clinical outcomes [14, 15] but also to reduced physician burnout, increased job satisfaction, and improved mental and physical well-being [16–18]. Importantly, clinical empathy among medical students has been consistently linked to their clinical competence rather than to academic or examination-based performance [19], underscoring its role as a professional and context-dependent capacity rather than a purely cognitive or academic attribute. Although the critical importance of empathy is widely recognized, its developmental trajectory throughout medical education remains unclear. Systematic reviews have synthesized approximately 60 empirical studies conducted between 1998 and 2019 across North America, Europe, and Asia. Taken together, these findings are inconsistent: 27 studies reported a decline in empathy during medical education, 20 reported no significant change, and 13 reported an increase. In contrast, among the 13 longitudinal studies, the results were more convergent, with 10 showing decreases, 2 remaining stable, and only 1 short-term study (1 year) reporting an increase [13, 20–22]. These discrepancies may partly reflect differences in study design but, more fundamentally, stem from variation in how empathy is conceptualized and measured—particularly whether assessments focus on relatively stable empathic traits or on context-sensitive expressions of clinical empathy. Longitudinal designs incorporating multiple empathy measures are therefore particularly valuable for disentangling these distinct aspects of empathy development.

In China, despite hosting the largest population of medical students worldwide, long-term longitudinal studies examining empathy development remain scarce. Guided by a trait–state and context-sensitive conceptual framework, the present study aims to address this gap. We employed a longitudinal design, following the same cohort of students enrolled in the five-year clinical medicine program over the course of five years. Both the JSE-S and the IRI were administered, and repeated-measures analyses were conducted to depict empathy trajectories and to explore potential demographic and educational predictors across the entire medical education cycle. This design allows us to examine whether clinical empathy and trait empathy follow distinct developmental trajectories over time. We hypothesized that: (1) clinical empathy measured by the JSE-S would exhibit significant changes across academic years, reflecting its sensitivity to educational and clinical contexts; (2) trait empathy measured by the IRI would remain relatively stable over time; and (3) individual factors such as age and career aspiration would show stronger associations with clinical empathy than with trait empathy. The findings are anticipated to enrich empirical evidence on empathy development in the Chinese context and to provide theoretical and practical insights for optimizing empathy cultivation in medical education.

## Methods

### Participants

A five-year prospective longitudinal study was conducted among clinical medicine students enrolled in a five-year undergraduate program at Fudan University was conducted. The initial data collection yielded 383 questionnaires. To ensure anonymity, identifiers were coded using the first three and last four digits of participants’ mobile phone numbers (e.g., 189****9292). After 11 questionnaires with incomplete identifiers were excluded, 372 valid responses remained.

For longitudinal analyses, only students who completed two or more survey waves were included (*n* = 104), resulting in a total of 266 valid questionnaires. Students who responded only once (*n* = 106) were excluded from the longitudinal modeling. Final analytic sample thus represented 49.5% of the participants with at least two valid responses (104/210) and accounted for 69.6% of all collected questionnaires (266/383).

Owing to staggered entry into the study, participants’ first responses occurred in different academic years, ranging from the first year to the fourth year. For each participant, the first available response was defined as the baseline, and subsequent responses were aligned accordingly for longitudinal analysis.

### Instruments

The self-administered questionnaire consisted of three sections:

- *Section A* collected demographic information. The selection of demographic variables in this study was guided by prior empirical literature demonstrating their associations with empathy among Chinese medical students [10, 12]. These studies consistently identified gender, student leadership role, career aspiration, and academic year as significant correlates of empathy in this population.
- *Section B* included the Chinese version of the Jefferson Scale of Empathy–Student version (JSE-S). The translation and cultural adaptation of this version followed a structured validity verification procedure: content validity was first established through expert review, followed by an assessment of face validity via informal cognitive interviews with medical students to confirm that the items were clear, relevant, and easy to answer. The scale comprises 20 items rated on a 7-point Likert scale (1 = strongly disagree to 7 = strongly agree). Half of the items were positively worded, and half were negatively worded. The total scores ranged from 20 to 140, with higher scores reflecting greater levels of clinical empathy. The JSE-S has demonstrated satisfactory psychometric reliability in previous studies [10, 12].
- *Section C* included the Chinese version of the Interpersonal Reactivity Index (IRI), which assesses general empathy. It contains 22 items rated on a 5-point Likert scale (1 = strongly disagree to 5 = strongly agree), with five items negatively worded. The total scores ranged from 22 to 110, with higher scores reflecting stronger dispositional empathy.

### Procedures

We recruited class advisors—faculty members at Chinese universities responsible for both academic guidance and student affairs—as data collectors. After standardized training to ensure procedural consistency, collectors distributed the online questionnaire via class WeChat groups and invited students to participate voluntarily during the same period each academic year. The first wave of data collection took place in October 2019, and the final wave was completed in October 2023.

An explanatory statement was provided at the beginning of the questionnaire, informing students that participation was voluntary and anonymous and that data would be reported in aggregate for research purposes only. Completion of the questionnaire was regarded as implied informed consent. The study protocol was approved by the Research Ethics Committee of the School of Basic Medical Sciences, Fudan University.

### Statistical analyses

All analyses were conducted using IBM SPSS Statistics version 20.0. Descriptive statistics were performed on baseline data (i.e., each participant’s first available response), and the internal consistency of the JSE-S and IRI was assessed via Cronbach’s α.

To evaluate changes in empathy over time and associated factors, a linear mixed-effects model was constructed with the JSE-S total score as the dependent variable. Fixed effects included categorical variables—academic year, age group, career aspiration, and entry year (i.e., the year in which a student first joined the study)—as well as the continuous covariates of the total IRI score. The entry year was modeled as a categorical factor to adjust for potential cohort-specific differences due to staggered enrollment, especially considering structural disruptions such as the COVID-19 pandemic.

Participant ID was included as a random effect to account for repeated measurements, with an autoregressive covariance structure [AR(1)] assumed. Type III sums of squares were used for hypothesis testing (α = 0.05).

A parallel model was constructed with the IRI total score as the dependent variable to examine predictors of trait empathy, using the same fixed and random-effects structure.

## Results

### Descriptive statistics

Baseline data, including data from 104 medical students, were obtained from the first measurement. Most participants were female (63.46%) and aged ≤ 19 years (82.69%). The majority were in their first (59.62%) or second (33.65%) year of study. A high proportion (88.46%) reported a willingness to pursue a medical career, and 45.19% held student leadership roles. Additionally, 58.65% were from one-child families. The participants’ hometowns spanned cities, towns, and rural areas (Table 1).

**Table 1:** Sample Characteristics at Baseline Measurement (*N* = 104)

Variable	Category	*n*	%
Gender	Male	38	36.54
	Female	66	63.46
Age group	≤ 19 years	86	82.69
	20–22 years	18	17.31
School year	1st year	62	59.62
	2nd year	35	33.65
	3rd year	4	3.85
	4th year	3	2.88
Student leadership role	Yes	47	45.19
	No	57	54.81
Only child status	Yes	61	58.65
	No	43	41.35
Hometown location	Municipality	19	18.27
	Provincial capital	16	15.38
	Small/medium city	30	28.85
	Town	23	22.12
	Rural area	16	15.4
Career aspiration: willingness to become a doctor	Willing	92	88.46
	Unwilling	1	0.96
	Undecided	11	10.58

Both the JSE-S and the IRI demonstrated good internal consistency, with Cronbach’s α values of 0.80 and 0.78 at baseline and 0.82 and 0.79 across all valid responses, respectively.

Across the full dataset, the JSE-S total scores ranged from 72 to 134 (theoretical range: 20–140), with a mean of 106.84 (SD = 11.89). The IRI scores ranged from 52 to 106, with a mean of 78.32 (SD = 8.80). The skewness and kurtosis values were − 0.12 and − 0.20 for JSE-S and − 0.03 and − 0.26 for IRI, respectively. All corresponding Z values were within ± 1.96, indicating approximate normal distributions for both scales. No outliers were detected.

### Model fit and fixed effects

A linear mixed-effects model was constructed with the JSE-S total score as the dependent variable. When an AR(1) covariance structure and maximum likelihood estimation were used, the model fit was good: −2 restricted log-likelihood = 1923.91; AIC = 1929.91; and BIC = 1940.51.

The covariance parameter estimates indicated an AR (1) diagonal variance of 81.21 (SE = 14.08), an autoregressive coefficient (rho) of 0.20 (SE = 0.17), and a subject-level random intercept variance of 25.70 (SE = 13.98).

Type III tests of fixed effects revealed that academic year (F = 4.68, *p* = .001), age group (F = 5.71, *p* = .004), career aspiration (F = 4.31, *p* = .014), and the IRI score (F = 25.77, *p* < .001) were significant predictors of JSE-S. Entry year was modeled as a categorical factor and did not have a significant main effect (F = 1.54, *p* = .207) (see Table 2).

**Table 2:** Tests of Fixed Effects in the Mixed Linear Model Predicting Empathy (JSE-S)

Predictor	df (num/den)	F	*p*
Academic year	4 / 170.60	4.68	0.001
Age group	2 / 229.70	5.71	0.004
Career aspiration	2 / 237.68	4.31	0.014
Entry year	3 / 112.72	1.54	0.207
IRI score	1 / 234.05	25.77	< 0.001

Note. Fixed effects were tested using Type III F-tests with numerator (num) and denominator (den) degrees of freedom

### Negative impact of academic year on JSE-S scores

**Fig. 1:**
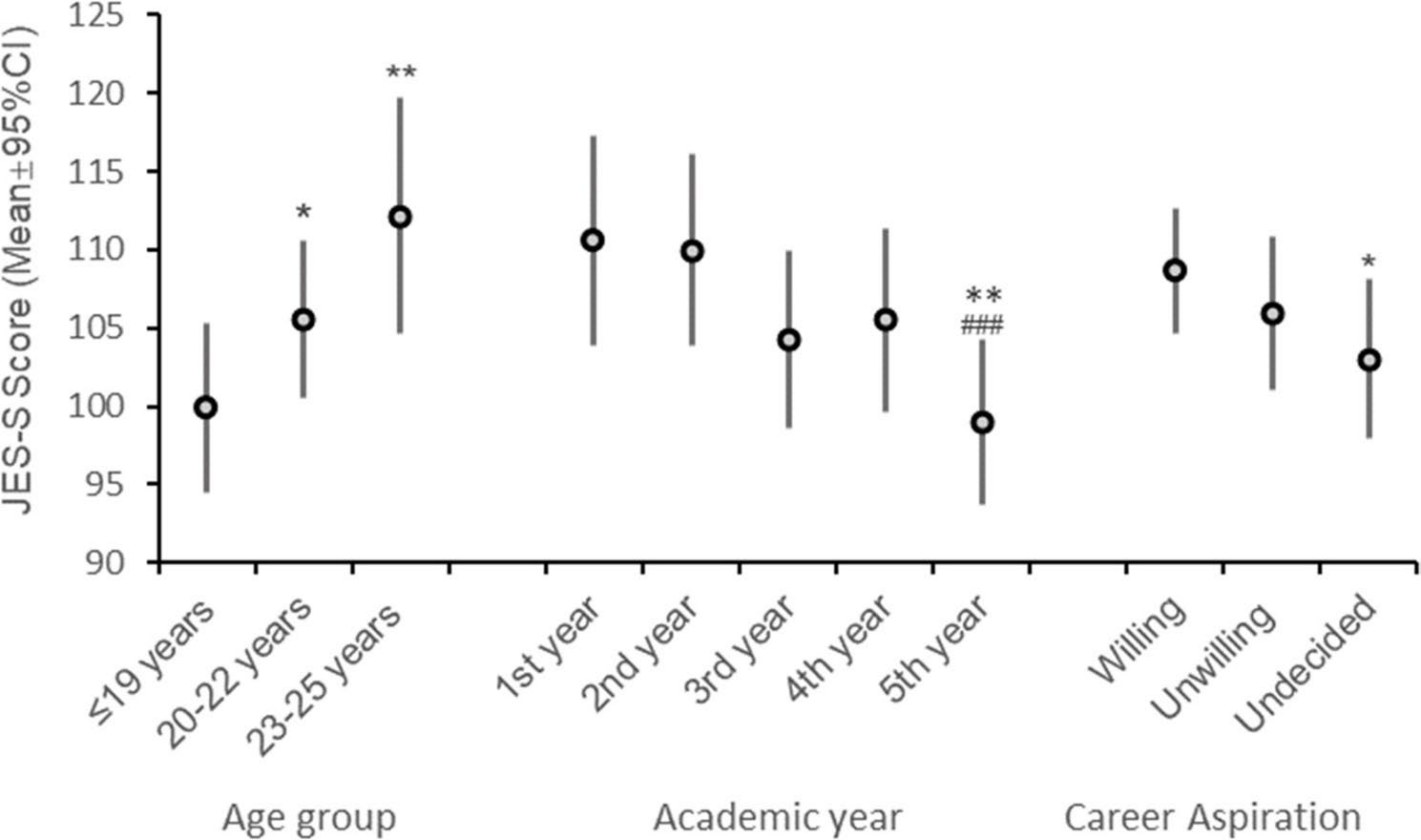
Adjusted Marginal Means of JSE-S Scores by Key Predictors. Legend: Marginal means derived from linear mixed models adjusted for the IRI (fixed at 78.32), with age, entry year, and gender held at sample means. The error bars represent 95% confidence intervals. Significance: *p < .05, **p < .01 vs. reference group (1st year/≤19 years/Willing); ### *p* < .001 vs. 2nd year

Controlling for IRI, age group, career aspiration, and entry year, academic year had a significant main effect on JSE-S scores (F (4, 170.60) = 4.68, *p* = .001). The empathy scores were highest in the first year and showed a general declining trend across subsequent academic years. Compared with those of first-year students, JSE-S scores were significantly lower in the third year (B = − 6.29, *p* = .019, 95% CI [− 11.55, − 1.04]) and in the fifth year (B = − 11.57, *p* < .001, 95% CI [− 17.69, − 5.56]) (see Table 3). Bonferroni-adjusted pairwise comparisons confirmed significant differences between the 5th and 1st years (*p* = .002) and between the 5th and 2nd years (*p* = .001). Differences between the 5th and 4th years (*p* = .075) and between the 3rd and 2nd years (*p* = .088) showed marginal significance (see Fig. 1).

**Table 3:** Fixed Effects Estimates from the Mixed Linear Model Predicting Empathy (JSE), Adjusted for IRI

Parameter	Estimate	SE	df	t	*p*	95% (CI)
Intercept	80.70	8.84	177.98	9.13	< 0.001	[63.25, 98.14]
IRI	0.40	0.08	234.05	5.01	< 0.001	[0.24, 0.55]
Academic year						
First year (ref.)	–	–	–	–	–	–
Second year	-0.59	1.55	141.68	-0.39	0.706	[-3.66, 2.49]
Third year	-6.29	2.67	224.89	-2.36	0.019	[-11.55, -1.04]
Fourth year	-5.03	3.11	219.72	-1.62	0.107	[-11.16, 1.10]
Fifth year	-11.57	3.05	227.30	-3.79	< 0.001	[-17.58, -5.56]
Age group						
≤ 19 years (ref.)	–	–	–	–	–	–
20–22 years	5.65	2.17	236.88	2.60	0.010	[1.36, 9.93]
23–25 years	12.27	3.70	250.99	3.21	0.001	[4.97, 19.56]
Career aspirations after graduation						
Willing to be a doctor(ref.)	–	–	–	–	–	–
Unwilling to be a doctor	-2.75	4.48	232.57	-0.62	0.539	[-11.57, 6.07]
Undecided	-5.67	1.93	242.80	-2.94	0.004	[-9.47, -1.87]

Note. Estimates are unstandardized regression coefficients (B) from the mixed linear model using maximum likelihood estimation with an AR(1) covariance structure

Reference groups are indicated as “ref.”

*SE* Standard error, *df* denominator degrees of freedom, *CI* Confidence interval

### Age-related increases in JSE-S scores

Older students reported significantly higher empathy scores (F (2, 229.71) = 5.71, *p* = .004). Compared with students aged ≤ 19 years, those aged 20–22 years scored higher (B = 5.65, *p* = .010, 95% CI [1.36, 9.93]), and those aged 23–25 years scored even higher (B = 12.27, *p* = .001, 95% CI [4.97, 19.56]) (see Table 3).

Bonferroni-adjusted comparisons revealed that both the 23–25 and 20–22 groups scored higher than ≤ 19 (*p* = .003 and *p* = .030, respectively). The 23–25 group also scored higher than the 20–22 group did, with marginal significance (*p* = .068) (see Fig. 1).

### Effects of career aspiration on JSE-S scores

Career aspiration significantly influenced JSE-S scores (F (2, 237.68) = 4.31, *p* = .014). Students who expressed a willingness to become doctors scored significantly higher than those who were uncertain did (B = − 5.67, *p* = .004, 95% CI [− 9.47, − 1.87]) (see Table 3).

### Positive predictive effect of IRI on JSE-S

**Fig. 2:**
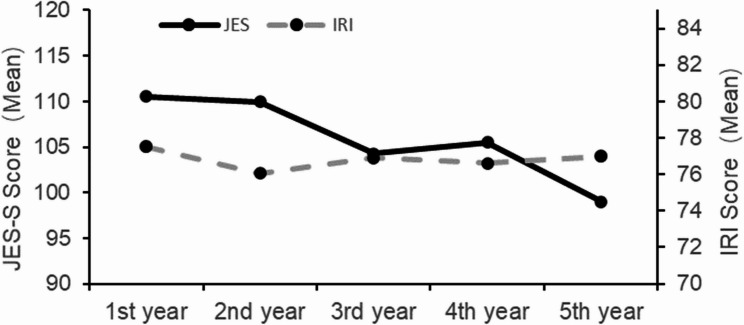
Trajectories of JSE-S and IRI scores over five years based on marginal estimates from linear mixed models. Legend: JSE-S scores significantly decreased across academic years (see Fig. 1), whereas IRI scores remained stable throughout the study period (all between-year comparisons: *p* > .05). All models were adjusted for baseline scores

Pearson correlation analysis revealed a moderate positive association between the JSE-S score and the IRI (*r* = .371, *p* < .001). In mixed linear models, IRI scores significantly predicted JSE-S (B = 0.40, *p* < .001) even after adjusting for age, grade, career aspiration, and entry year. However, the IRI scores remained stable across academic years (F (4, 171.34) = 0.57, *p* = .685), with no significant associations for age group, career aspiration, or entry year in the adjusted models. This stability contrasts with the significant longitudinal decline in JSE-S scores (see Fig. 2).

## Discussion

To our knowledge, this study is among the first longitudinal investigations in China to examine the trajectory of empathy among undergraduate medical students. Our findings revealed a general downward trend in context-specific clinical empathy, as measured by the JSE-S, over the five years of medical education. In contrast, scores on the IRI, which reflect stable empathic traits, remained relatively constant throughout the study period. Additionally, older students were found to score higher on the JSE-S, suggesting that age has a positive association with clinical empathy. Career aspiration was also a significant predictor of empathy: students who were undecided about pursuing a medical career had significantly lower JSE-S scores than those who clearly expressed their willingness to become doctors. Taken together, these findings largely support the study hypotheses, confirming divergent longitudinal trajectories of trait and clinical empathy and highlighting the role of dispositional empathy and career motivation in shaping clinical empathic expression. Overall, these patterns are consistent with a trait–state conceptualization of empathy, in which relatively stable dispositional empathy provides a general capacity, while clinical empathy reflects a context-sensitive expression shaped by educational and motivational factors.

In China, the five-year clinical medicine program typically follows a “4 + 1” training model, consisting of four years of coursework followed by one year of clinical internship. At Fudan University, a comprehensive university [23], the medical school curriculum is structured as follows: The first year mainly consists of general education and foundational science courses with limited medical content. The second year focuses on basic medical sciences. The first semester of the third year continues with basic medical sciences (accounting for three-quarters of weekly contact hours), whereas the second semester marks a full transition to clinical coursework and incorporates bedside teaching. The fourth year continues with clinical coursework and clerkships, and the fifth year consists of full-time clinical internships with rotations through multiple hospital departments. Given the limited clinical exposure of students in the early years, this study employed both the JSE-S, which assesses context-specific empathy in healthcare settings, and the IRI, a generic empathy scale, to provide a comprehensive evaluation of medical students’ empathy. The use of dual instruments helped offset the limitations of using a single scale and minimized the interpretive bias caused by differing measurement emphases.

A key finding of this study is the divergent trajectories of the two empathy measures: JSE-S scores showed an overall declining trend across academic years, reaching their lowest point in the fifth year during the internship, whereas IRI scores remained stable throughout the same period. This discrepancy may reflect fundamental differences in the measurement focus of the two scales [4]. The IRI captures trait empathy, a relatively stable personality disposition shaped by long-term life experiences, whereas the JSE-S assesses context specific, clinical empathy, particularly the cognitive and behavioral aspects of physician–patient interactions, which are subject to situational modulation. Our findings suggest that while dispositional empathy remained relatively intact, students’ reported expression of empathy in clinical contexts diminished over time. This pattern underscores that empathy is not merely a stable personal disposition but a context-sensitive construct, in which relatively stable cognitive–affective capacities (trait empathy) interact with situational demands, emotional regulation, and professional expectations to shape clinical empathic expression.

This distinction is also supported by the literature. Studies using the IRI have often reported stable [24–26] or only minimally varying empathy levels [27] across medical education and low sensitivity to educational interventions [28]. In contrast, JSE scores are more responsive to contextual factors such as curriculum design, patient expectations, and cultural factors [29], exhibiting greater malleability in response to educational interventions [30]. For example, educational interventions aimed at enhancing empathy often yield significant improvements in JSE scores but produce negligible [31] or no change [30] in IRI scores.

Our findings are consistent with those of prior studies in China that documented a decline rather than an increase in empathy among medical students [32, 33]. Similar trends have been observed in other countries, including the United States and Iran [34, 35]. This decline aligns with our previous cross-sectional study of medical students in an eight-year program, which revealed a significant decrease in empathy during the internship phase. Several factors may contribute to this decline. First, upon transitioning to clinical rotations, students are immersed in a high-intensity, high-workload clinical environment that may compel them to prioritize technical tasks, reducing opportunities for physician‒patient communication. This results in a task-oriented approach overshadowing humanistic care, potentially reducing opportunities for empathic engagement [12, 36]. Second, students may resort to psychological self-protection strategies such as “dehumanizing patients” to shield themselves from the emotional burden associated with pain, suffering, and death, consequently attenuating empathic expression [20]. Third, structural limitations in medical education may also contribute: current admission criteria emphasize standardized academic examination scores with limited attention to humanistic attributes. Once enrolled, the curriculum often prioritizes biomedical knowledge and technical competencies over the cultivation of emotional competence. Upon entering clinical settings, a hospital culture dominated by professional detachment, technological orientation, and efficiency-first principles may further hinder the development of empathy [36]. These factors may be understood as contextual pressures that selectively constrain the expression of state-level clinical empathy, rather than eroding underlying dispositional empathic capacity.

In contrast, trait empathy, as measured by the IRI, a dispositional and personality-based construct, appears more resistant to contextual factors such as professional role expectations and curricular structures, showing high cross-situational stability throughout medical education. Our data further revealed a significant positive correlation between the IRI and JSE scores: each one-point increase in the IRI was associated with an average increase of 0.4 points in the JSE. This suggests that although trait empathy remains stable, it continues to play a facilitating role in students’ context-specific empathic expression in clinical settings, consistent with a trait–state interaction perspective.

We also found that age was a significant positive predictor of JSE scores: older students scored significantly higher than younger students did, whereas IRI scores remained stable across age groups. Previous research indicates that affective empathy tends to increase with age before adulthood [37], whereas cognitive empathy becomes relatively stable after individuals enter adulthood and only begins to decline after approximately 65 years of age [38]. Both affective and cognitive empathy continue to vary across the lifespan, suggesting that state measures of empathy are particularly susceptible to context-dependent fluctuations [39]. This perspective helps explain why the IRI, as a measure of trait empathy, demonstrated stability across age groups among adult medical students, whereas the JSE, which focuses on context-specific clinical expression, exhibited age-related score differences. Supporting this interpretation, van de Graaff et al. reported no significant age differences in IRI scores among adolescents aged 16–20, yet task-induced state empathy measures revealed higher empathy levels in older participants (18–20 years) [39]. These findings further support the notion that trait empathy remains relatively stable with age, while state empathy retains developmental plasticity.

Importantly, although age had a positive effect on JSE scores, the overall trend still showed a decline across academic years. This finding further supports the central role of context-related factors in shaping empathy development. As students progress through medical school, increasing academic demands, pressures related to postgraduate advancement and career preparation, and increasing clinical challenges may collectively erode their capacity for context-specific empathic expression in clinical settings. In other words, the potentially beneficial effects of age on empathy may be attenuated or even overridden by the modulatory influences of the educational and clinical environment, thereby contributing to the observed downward trend in JSE scores. From this perspective, the relationship between age and empathy should be understood as a contextually modulated, multidirectional pattern whose manifestations are jointly shaped by factors such as the type of measurement tool, the empathy dimension assessed, and the educational environment. This may help explain the inconsistent findings regarding age-related empathy reported in the literature [32, 40, 41].

Our previous cross-sectional studies revealed a strong association between medical students’ career aspirations and their empathy levels [10, 12]. Specifically, students who aspired to pursue a medical career after graduation consistently presented higher JSE scores than those who were uncertain or did not plan to pursue medical careers. The present longitudinal findings are consistent with earlier results, further confirming that career aspiration is a key determinant of JSE scores. Mixed linear model analyses revealed that students who expressed a willingness to become doctors had significantly higher JSE scores than those who were undecided did, with a mean difference of 5.67 points (β = -5.67, *p* = .004). Although no significant difference in JSE scores was observed between the two groups at baseline (t (101) = -1.28, *p* = .203; Cohen’s d = -0.41) and both groups exhibited similar rates of decline over time (time × career interaction effect, *P* > .05), students intending to pursue a medical career consistently demonstrated higher JSE scores throughout their medical education. This difference is unlikely to stem from divergent trajectories of empathy development but rather suggests that the divergence emerged and was sustained during the course of medical education.

Within a trait–state and context-sensitive framework, professional identity formation (PIF) may represent a theoretically plausible pathway through which career aspiration could be associated with sustained levels of clinical empathic expression. Given the documented associations among career aspiration, professional identity formation (PIF), and empathy [42], PIF represents a potentially relevant theoretical framework through which the observed relationship may be interpreted. However, because PIF was not directly measured in the present study, its role should be considered a hypothesis for future research rather than a mechanism supported by the current data. PIF involves both the internalization of professional roles, values, and behaviors and the integration of individual characteristics with the expectations of the medical profession. Empathy acts as a key bridge in this process of integration and internalization [43]. Students with a clear intention to pursue a medical career may therefore be more likely to develop a more stable professional identity, which could be associated with relatively higher levels of clinical empathic expression over time. Although the JSE scores showed an overall decline during medical education, career-committed students consistently demonstrated higher JSE scores, a pattern that may be conceptually consistent with this perspective. From a motivated behavior perspective, these findings suggest that career aspiration may function as a sustained motivational factor that supports the enactment of empathic behaviors in clinical contexts, even in the presence of increasing educational and emotional demands. Future studies should incorporate validated PIF assessment tools to explicitly examine this hypothesized pathway and to clarify the role of professional identity formation in empathy development during medical training.

### Limitations

Despite the strengths of its five-year longitudinal design and the use of dual measurement tools, this study has several limitations. First, the sample was drawn from a single university, which may limit the generalizability of the findings. Second, both the JSE-S and the IRI are self-reported measures that assess empathy-related attitudes rather than actual behaviors and are subject to social desirability bias. Future studies should complement self-reports with additional methods such as direct observation, peer evaluations, and feedback from standardized patients. Third, this study followed students only through the first semester of their final year, excluding the complete internship period. Since empathy — and related aspects of professional development — continue to evolve during clinical training, extending the observation period through graduation would provide a more complete view of the developmental trajectory. Finally, the study period (2019–2023) overlapped with the COVID-19 pandemic, which may have influenced medical training contexts; therefore, the findings should be interpreted and generalized to non-pandemic settings with caution.

## Conclusion

This study is the first longitudinal investigation of empathy development among Chinese undergraduate medical students. The findings revealed a general decline in context-specific clinical empathy (JSE) over five years, reaching its lowest point during the internship, whereas trait-based empathy (IRI) remained relatively stable. This divergence underscores the important role of educational context in shaping the expression of clinical empathy. Age positively predicted JSE scores, which is conceptually consistent with theories of multidirectional age-related empathy development. Moreover, career aspiration emerged as an important factor, with students who clearly intended to become doctors consistently achieving higher JSE scores. These results indicate that empathy is shaped by both individual dispositions and educational exposures. Conceptually, the findings support a trait–state and context-sensitive understanding of empathy, highlighting how cognitive–affective dispositions, motivational factors, and educational environments jointly influence the development and expression of clinical empathy during medical training. Medical educators should therefore prioritize curriculum design, learning environments, and clinical training strategies that explicitly foster the sustained development of empathy throughout medical education.

## Data Availability

The data presented in this manuscript have not been published elsewhere. Data from this project will not be shared. Consent was not sought from participants to share the data more widely than for this study.

## Abbreviations

JSE-S: Student Version of the Jefferson Scale of Empathy
IRI: Interpersonal Reactivity Index
PIF: Professional Identity Formation
